# Periodic expression of *Per1* gene is restored in chipmunk liver during interbout arousal in mammalian hibernation

**DOI:** 10.1038/s41598-025-87299-8

**Published:** 2025-02-13

**Authors:** Erina Nakamaru, Kota Seki, Yuiho Shirahata, Megumi Adachi, Nene Sakabe, Takuya Matsuo, Daisuke Tsukamoto, Nobuhiko Takamatsu

**Affiliations:** Laboratory of Molecular Biology, Department of Biosciences, School of Science, Kitasato University, 1-15-1 Kitazato, Minami-ku, Sagamihara-shi, Kanagawa 252-0373 Japan

**Keywords:** Circadian rhythm, Clock gene, *Per1*, Periodic expression, Transcription regulation, Mammalian hibernation, Gene regulation, Transcription, Animal behaviour, Animal physiology

## Abstract

Circadian rhythms play an important role in many physiological processes. We have previously reported that no periodic fluctuation in the *Bmal1* mRNA is observed in the liver of the chipmunk, a mammalian hibernator, in the hibernation season, suggesting that peripheral circadian clocks are not functional during hibernation. In contrast, the *Per2* mRNA levels are transiently increased by elevated body temperature during interbout arousal and showed periodic fluctuations in the hibernation season, suggesting that periodic expression of the *Per2* mRNA may be restored during interbout arousal. In the present study, we analyzed *Per1* gene expression in the chipmunk liver. The *Per1* mRNA showed circadian fluctuations with a peak during the late sleep period in the non-hibernation season and periodic fluctuations with a peak during the early interbout arousal in the hibernation season. In both the non-hibernation and hibernation seasons, *Per1* gene expression was phase-advanced relative to *Per2* gene expression, and the phase relationship between the two genes was maintained, suggesting that for some genes, periodic gene expression, similar to circadian expression in the non-hibernation season, may be restored during interbout arousal. Interestingly, *Per1* gene transcription was differentially activated by BMAL1 in the non-hibernation season and possibly by CREB1 in the hibernation season.

**Supplementary Information:**

The online version contains supplementary material available at 10.1038/s41598-025-87299-8.

## Introduction

Almost all living organisms on Earth have a circadian rhythm with a cycle of about 24 h. Circadian rhythm is a physiological function that organisms have evolved to adapt to the periodic changes in light and dark associated with the Earth’s rotation. In mammals, circadian rhythms are observed in various physiological phenomena such as the sleep-wake cycle, body temperature (Tb), and blood pressure^1^. Circadian rhythms are regulated by hierarchically organized body clocks consisting of a master clock located in the suprachiasmatic nucleus (SCN) of the hypothalamus and slave clocks in the periphery (peripheral clocks)^2^. At the core of the molecular machinery of the circadian clock is a transcriptional-translational feedback loop (TTFL) formed by the core clock genes *Bmal1*, *Clock*, *Cryptochrome* (*Cry1*, *Cry2*), and *Period* (*Per1*, *Per2*, *Per3*)^3^. BMAL1-CLOCK heterodimers activate the transcription of the *Pe*r and *Cry* genes via E-box enhancer elements, and subsequently, the PER-CRY complexes inhibit the transcriptional activity of BMAL1-CLOCK. Once the PER and CRY levels drop sufficiently, CLOCK-BMAL1-mediated transcription can resume and a new oscillatory cycle begins. One of the characteristics of the circadian clock is its ability to synchronize with daily changes in the environment. Since the cycle length of the circadian rhythm is not exactly 24 h, it is necessary to adjust the phase of the circadian rhythm to synchronize it with the 24 h light-dark cycle based on the Earth’s rotation. The most powerful entrainment cue for circadian rhythms is light. The SCN is the only clock that is reset by light from the environment, and the photic signals are transmitted to the SCN via the retinohypothalamic tract^4–6^. In the SCN, cAMP response element-binding protein (CREB) binds to the CREB binding site (CRE, cAMP response element) upstream of the *Per1* gene, and transcription of the *Per1* gene is transiently activated, resulting in light-induced resetting of the circadian clock^7,8^. Although light signals also induce the expression of the *Per2* gene, the *Per2* gene is less inducible than the *Per1* gene^9^, and the CRE in the *Per1* gene promoter is not involved in light induction^10^. Another property of the circadian clock is temperature compensation, which allows organisms to keep the period of the circadian rhythm relatively constant over a wide range of physiological temperatures^11^.

Certain small mammals hibernate to survive the winter season when environmental temperatures drop and food is scarce. In the hibernation (winter) season, these mammalian hibernators regularly undergo cycles of deep torpor, during which their Tb is only a few degrees above ambient temperature, and interbout arousal, during which their Tb rises to the normothermic levels^12^. Although hypothermia occurs during deep torpor, the regularity of the deep torpor-interbout arousal cycle and the temperature-compensating nature of circadian rhythms have led to research into whether the circadian clock functions during deep torpor, and several papers have been published both supporting and denying that the circadian rhythm functions during deep torpor^13–21^. Analyses of the temporal variation in the expression of the clock genes in the SCN showed no variation in expression^18,21^. Furthermore, when the circadian rhythms of the SCN neurons were monitored in mouse and Syrian hamster SCN slices, the circadian rhythms of *Baml1* and *Per2* expression were maintained at 22 °C, but suspended at 15 °C^22^. These results strongly support the idea that the circadian clock stops functioning during deep torpor.

The chipmunk (*Tamias asiaticus*) is an obligate hibernator belonging to the family Sciuridae. When kept in constant darkness at 4 °C in the hibernation season, chipmunks alternate between 5−6 days of deep torpor with a Tb of ~ 5–7 °C and ~ 20 h of interbout arousal^23,24^. To investigate whether the peripheral circadian clock functions in the hibernation season, we analyzed the expression of the clock genes *Bmal1* and *Per2* in the chipmunk liver^25^. The results showed that there was no periodicity in *Bmal1* mRNA fluctuations in the hibernation season, suggesting that the clock gene TTFL was not formed and the peripheral circadian clock was not functioning. On the other hand, the *Per2* mRNA remained at low levels during deep torpor, consistent with a previous report that transcription and translation are globally suppressed during deep torpor^26^. Notably, the *Per2* gene was transiently activated by heat shock factor 1 (HSF1), which was activated by elevated Tb during interbout arousal, resulting in periodic fluctuations in the *Per2* mRNA in the hibernation season. Periodic fluctuations in the hibernation season were also observed for *Rev-erba* mRNA, but not for *Dbp* mRNA^25^. The transcriptional activation of the *Per2* gene by HSF1 contributes to the synchronization of the peripheral clock with the Tb rhythm^27–30^. Therefore, the lack of resetting of the peripheral circadian clock in the hibernation season may be due to differences in the time required to reset each clock gene. When a temperature rhythm similar to the body temperature rhythm was applied to cultured cells, the *Per2* gene synchronized with the temperature rhythm immediately, but the *Bmal1* and *Dbp* genes took several days to synchronize^30^. Taken together, it is possible that the periodic expression of the *Bmal1* and *Dbp* genes is not restored because the interbout arousal is as short as 20 h. In the present study, to further explore the possibility that periodic gene expression is restored in a subset of genes during interbout arousal, we analyzed *Per1* gene expression in the chipmunk liver, since the regulation of the *Per1* and *Per2* genes may differ in peripheral tissues and cultured cells^31^. In the mouse liver, the *Per1* gene shows a circadian expression that is phase-advanced relative to that of the *Per2* gene^32^ and is unlikely to be transcriptionally activated by HSF1, which is activated by the Tb rhythm.

## Results

### *Per1* mRNA shows periodic fluctuations in the hibernation season

**Fig. 1:**
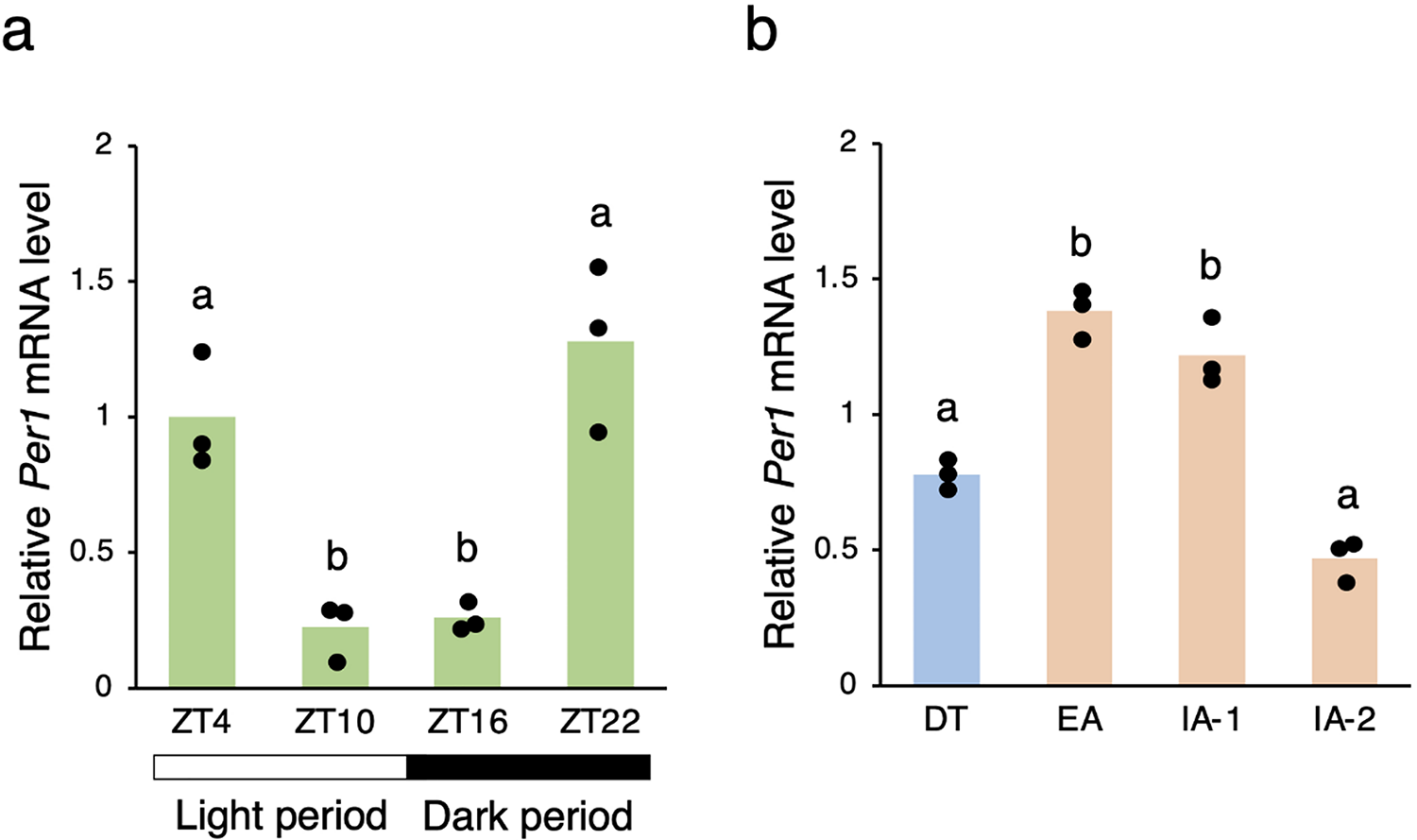
RT-qPCR analysis of* Per1* mRNA in chipmunk liver. The *Per1* and *Gapdh* mRNA levels in the non-hibernation (**a**) and hibernation (**b**) seasons were measured using chipmunk liver total RNA by RT-qPCR. qPCR was performed in triplicate. For each time point, three chipmunks were used for quantification. The *Per1* mRNA levels are normalized to the corresponding *Gapdh* mRNA levels and shown relative to the value obtained for ZT4. The data represent the mean and the scatter plots correspond to the observed values (*n* = 3/time point). Different letters indicate significant differences between different time points (Tukey’s HSD test, *p* < 0.05) and the same letters indicate insignificant differences (Tukey’s HSD test, *p*$\:\ge\:$ 0.05) (Supplementary Table S1).

We first investigated *Per1* mRNA expression in the liver of non-hibernating chipmunks. Chipmunks were kept at 23 °C on a 12 h–12 h light-dark cycle, with light on at Zeitgeber time (ZT) 0 and off at ZT12. *Per1* mRNA expression was analyzed by RT-qPCR every 6 h at ZT4, ZT10, ZT16 and ZT22 (Fig. 1a). The *Per1* mRNA levels showed a peak at ZT22 during the sleep period and a trough at ZT10 during the wake period. Although the chipmunk is diurnal in the non-hibernation season, this circadian expression of the *Per1* mRNA during the sleep-wake cycle is consistent with the expression of the *Per1* mRNA in the liver of the nocturnal mouse^32^.

In the hibernation season, chipmunks were kept in constant darkness at 4 °C and regularly repeated 5–6 days of deep torpor and ~ 20 h of interbout arousal. The RT-qPCR analysis revealed that the *Per1* mRNA levels increased transiently in early arousal animals whose Tb had not reached 30 °C during rewarming after spontaneous arousal from torpor (EA), compared to deeply torpid animals with Tb of 5 to 7 °C (DT), showed a decreasing trend already in interbout awake animals with Tb of 30 to 35 °C (IA-1), and further decreased in interbout awake animals with Tb of 35 to 37 °C (IA-2) (Fig. 1b). Thus, the *Per1* mRNA levels in the liver, as well as the *Per2* mRNA levels^25^, fluctuated regularly in the torpor-arousal cycle throughout the hibernation season under a constant condition of 4 °C in darkness.

### *Per1* transcription is activated by BMAL1 in non-hibernation season

**Fig. 2:**
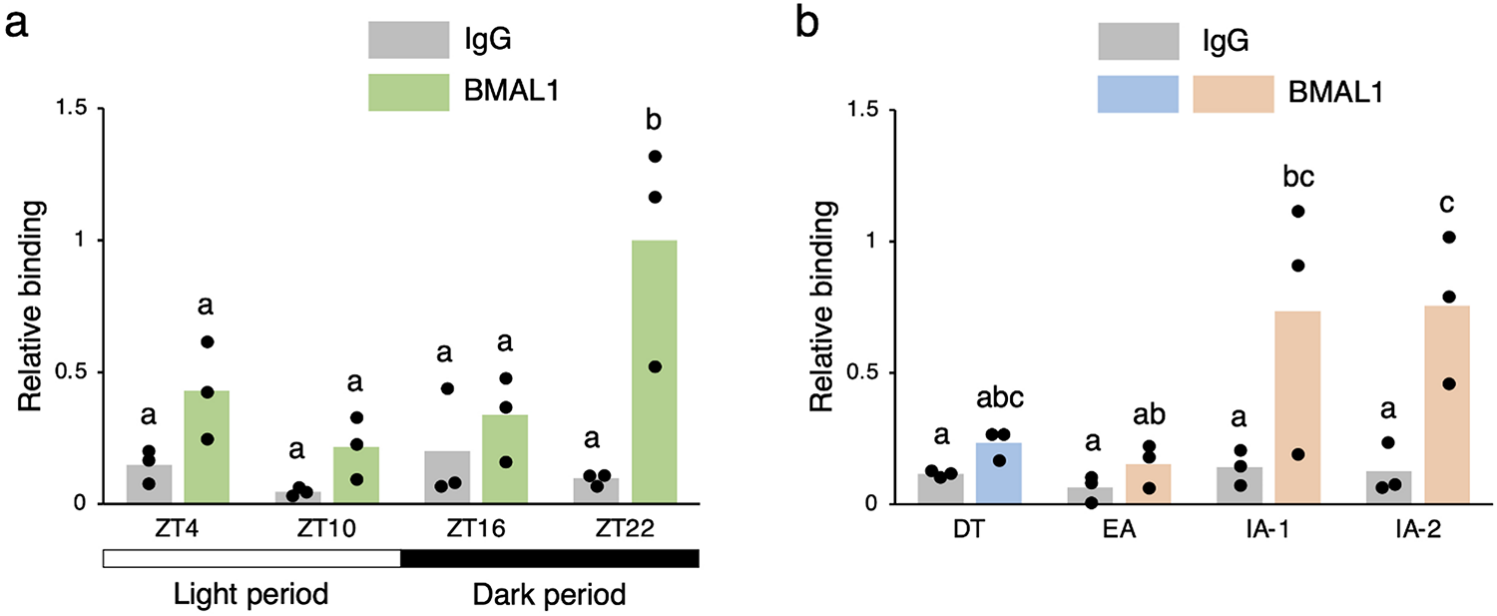
Binding of BMAL1 to 5′ flanking region of *Per1* gene in chipmunk liver. ChIP analysis was performed using antibodies against BMAL1 or histone H3 and normal rabbit IgG with chromatin prepared from the chipmunk liver. qPCR was performed in triplicate using a primer set specific for E-box at − 151 of the *Per1* gene (Supplementary Fig. S1). Normal IgG was used as a negative control. For each time point, three chipmunks were used for quantification. Values were normalized to the corresponding histone H3 values and shown relative to the value obtained for ZT22 with antibodies against BMAL1. The data represent the mean and the scatter plots correspond to the observed values (*n* = 3/time point). Different letters indicate significant differences between different time points (Tukey’s HSD test, *p* < 0.05) (Supplementary Table S2).

To investigate the transcriptional regulation of the chipmunk *Per1* gene, we isolated a genomic DNA fragment containing the promoter region of the chipmunk *Per1* gene based on the structure of the mouse *Per1* gene^33^. Approximately 1.9 kb of the 5′ flanking region of exon 1 of the chipmunk *Per1* gene contained a CRE and three E-boxes (Supplementary Fig. S1), the positions of which were well conserved with those in the promoter region of the mouse *Per1* gene^33^. Transcription of the *Per1* gene is activated by heterodimers of the clock proteins BMAL1 and CLOCK or NPAS2 via E-box sequences in the promoter region^33–35^. In the chipmunk liver, the *Bmal1* mRNA shows circadian fluctuations in expression with a peak at ZT16 and a trough at ZT4 in the non-hibernation season, but no periodic fluctuations in the hibernation season^25^. To investigate whether the heterodimers of BMAL1 and CLOCK or NPAS2 are involved in the transcriptional activation of the *Per1* gene in the chipmunk liver, we analyzed the binding of BMAL1 to the proximal E-box at – 151 in the 5′ flanking region of exon 1 of the *Per1* gene by chromatin immunoprecipitation (ChIP), since in the case of the mouse *Per1* gene, the proximal E-box is involved in the transcriptional activation by BMAL1-CLOCK^33^. In the non-hibernation season, the amount of BMAL1 bound to the E-box in the *Per1* gene promoter increased at ZT22 (Fig. 2a), consistent with the fluctuations in the *Per1* mRNA (Fig. 1a), suggesting that BMAL1-CLOCK or NPAS2 is involved in the transcriptional activation of the *Per1* gene. In the hibernation season, the *Per1* mRNA levels increased in EA (Fig. 1b), but BMAL1 binding to the *Per1* gene promoter was low in EA as well as in DT (Fig. 2b). These results suggest that, in contrast to the non-hibernation season, transcription factors other than BMAL1-CLOCK or NPAS2 are involved in the transcriptional activation of the *Per1* gene in EA.

### Two *Creb1* variants are expressed in chipmunk liver

**Fig. 3:**
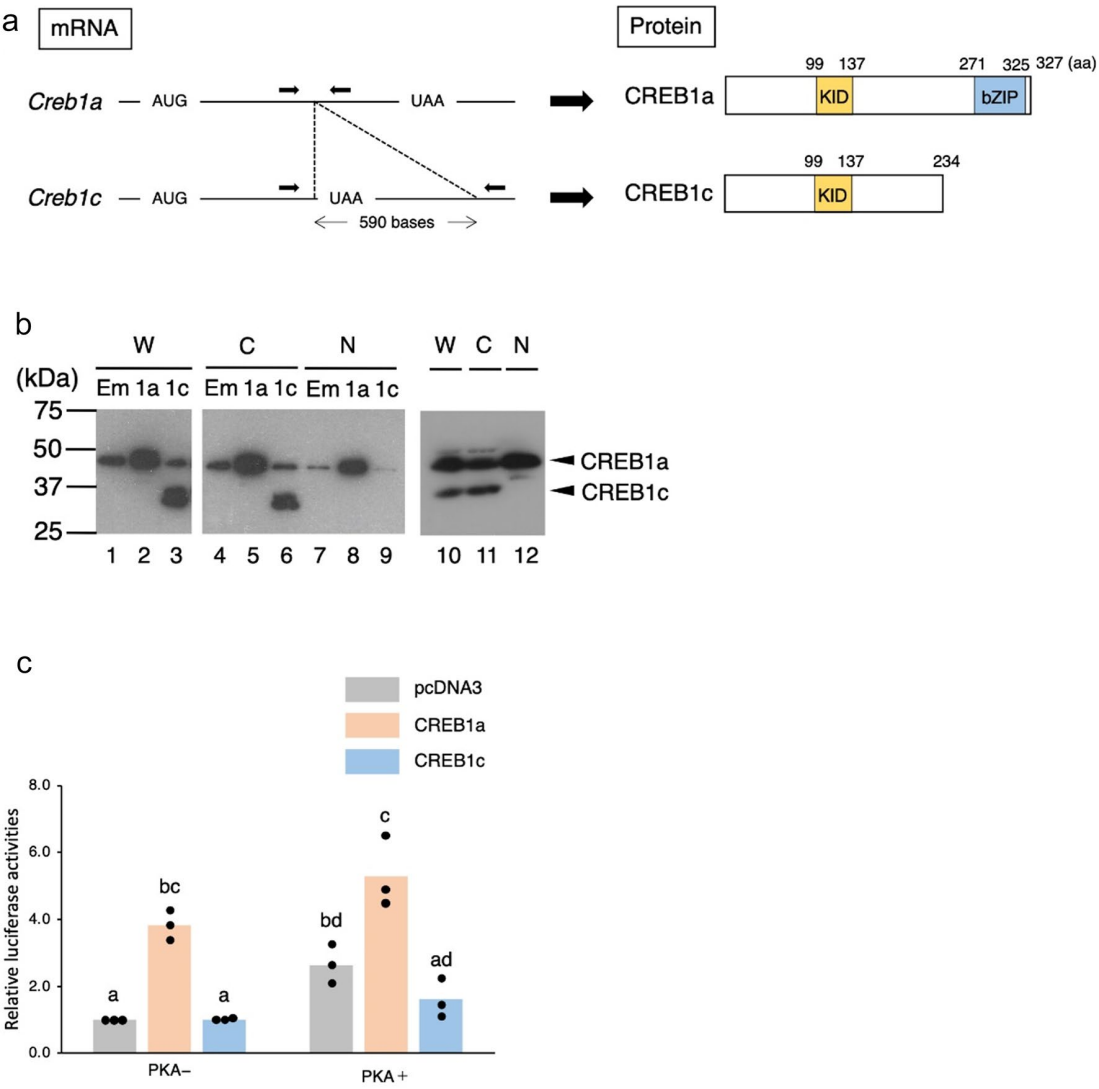
Analysis of *Creb1* variants expressed in chipmunk liver. (**a**) A schematic representation of the mRNA (left) and the protein (right) structures of the *Creb1* variants is shown. AUG and UAA indicate the translation initiation and termination codons, respectively. Arrows indicate the positions of the PCR primers used for the analysis of the ratio of the *Creb1c* mRNA to the *Creb1a* mRNA. The numbers above the boxes indicate the amino acid residues. (**b**) Western blot analysis was performed using whole cell extracts (W), cytoplasmic extracts (C), and nuclear extracts (N) prepared from 293T cells transfected with pcDNA3 (Em), pcDNA3/cmCREB1a (1a), or pcDNA3/cmCREB1c (1c) (lanes 1–9), or from the liver of a chipmunk in the non-hibernation season (lanes 10–12). (**c**) HepG2 cells were transfected with the chipmunk *Per1* gene luciferase reporter plasmid, pCMPER1G/luc, together with a *Renilla* luciferase plasmid pRL-SV40 as a control for transfection efficiency. Where indicated, the cells were co-transfected with pcDNA3, pcDNA3/cmCREB1a, or pcDNA3/cmCREB1c, and with or without the expression vector for the catalytic subunit of PKA, pFC-PKA. Each firefly luciferase activity was normalized to the *Renilla* luciferase activity and expressed as fold increase over that of pcDNA3 without pFC-PKA. The data represent mean from three independent experiments performed in triplicate and the scatter plots correspond to the observed values (*n* = 3). Different letters indicate significantly different values (Tukey’s HSD test, *p* < 0.05) (Supplementary Table S3).

Transcriptional activation of the *Per1* gene by CREB1 is involved in the light-induced resetting of circadian rhythms in SCN^8^. Since a CRE is present in the 5′ flanking region of the chipmunk *Per1* gene (Supplementary Fig. S1), to investigate whether CREB1 is involved in the transcriptional activation of the *Per1* gene in EA in the hibernation season, we isolated cDNA clones to the *Creb1* mRNA expressed in the chipmunk liver by PCR using primers designed on the basis of the ground squirrel *Creb1* mRNA that could amplify the entire protein coding region. As a result, two *Creb1* cDNAs corresponding to the human *CREB1* variant 6 (NM_001371427.1) encoding CREB1 isoform A (NP_001358356.1) and variant 3 (NM_001320793.2) encoding isoform C (NP_001307722.1) were isolated and named *Creb1a* and *Creb1c*, respectively (Fig. 3a). Compared to the *Creb1a* mRNA, the *Creb1c* mRNA has an insertion of 590 bases, suggesting that the two types of mRNA are thought to be produced by alternative splicing. The *Creb1a* mRNA encoded a protein of 327 amino acid residues (CREB1a) with a KID (kinase-inducible domain) and a bZIP DNA-binding domain, and its amino acid sequence was completely identical to that of human CREB1 isoform A. In contrast, the *Creb1c* mRNA encoded a protein of 234 amino acid residues (CREB1c) with only one KID domain, which differed from human CREB1 isoform C by one amino acid (M234L).

Since CREB1c lacks the bZIP domain (Fig. 3a), to investigate whether there are differences in the subcellular localization of CREB1a and CREB1c, HEK293T cells were transfected with an expression vector for CREB1a (pcDNA3/cmCREB1a) or CREB1c (pcDNA3/cmCREB1c), and whole cell extracts, cytoplasmic extracts, and nuclear extracts were prepared and subjected to Western blotting (Fig. 3b). The results showed that CREB1a was present in the nucleus and cytoplasm (lanes 5 and 8), whereas CREB1c was localized only in the cytoplasm (lanes 6 and 9). The same results were obtained in an analysis using extracts prepared from the chipmunk liver: endogenous CREB1a was found to be present in the nucleus and cytoplasm, whereas endogenous CREB1c was localized only in the cytoplasm (lanes 10–12).

To investigate the transcriptional regulation of the chipmunk *Per1* gene by CREB1, since a CRE is located at – 1698 in the 5ʹ flanking region of the chipmunk *Per1* gene, a reporter plasmid was created by fusing a sequence from − 1885 in the 5ʹ flanking region of exon 1 to + 1664 in the upstream of the translation initiation codon of exon 2 of the chipmunk *Per1* gene to the upstream of the luciferase gene in pGV-B (Supplementary Fig. S1). This reporter plasmid was transfected into HepG2 cells together with pcDNA3/cmCREB1a or pcDNA3/cmCREB1c, and luciferase activity was measured (Fig. 3c). In addition, CREB1 is activated by phosphorylation at the serine residue in the KID domain (serine 119 in the case of chipmunk CREB1a) by several kinases, including protein kinase A (PKA) and Ca^2+^/calmodulin-dependent protein kinase (CaMK)^36,37^. Since endogenous kinases that phosphorylate CREB1 in chipmunk liver have not yet been identified, to investigate the effect of CREB1 phosphorylation on *Per1* gene transcription, we used an expression vector for the catalytic subunit of PKA as a kinase capable of phosphorylating CREB1 where indicated (Fig. 3c). The results showed that transcription of the *Per1* gene was activated by CREB1a and was further enhanced by the phosphorylation of CREB1a. Luciferase activity was also increased by the addition of PKA when the empty vector pcDNA3 was transfected, suggesting that phosphorylation of endogenous CREB1 in HepG2 enhanced transcriptional activation of the *Per1* gene. In contrast, transfection of CREB1c alone did not alter luciferase activity, but the addition of PKA reduced activity compared to pcDNA3, suggesting that CREB1c has a repressive effect on the transcriptional activation by CREB1a.

### Analysis of *Creb1* gene expression in chipmunk liver

**Fig. 4:**
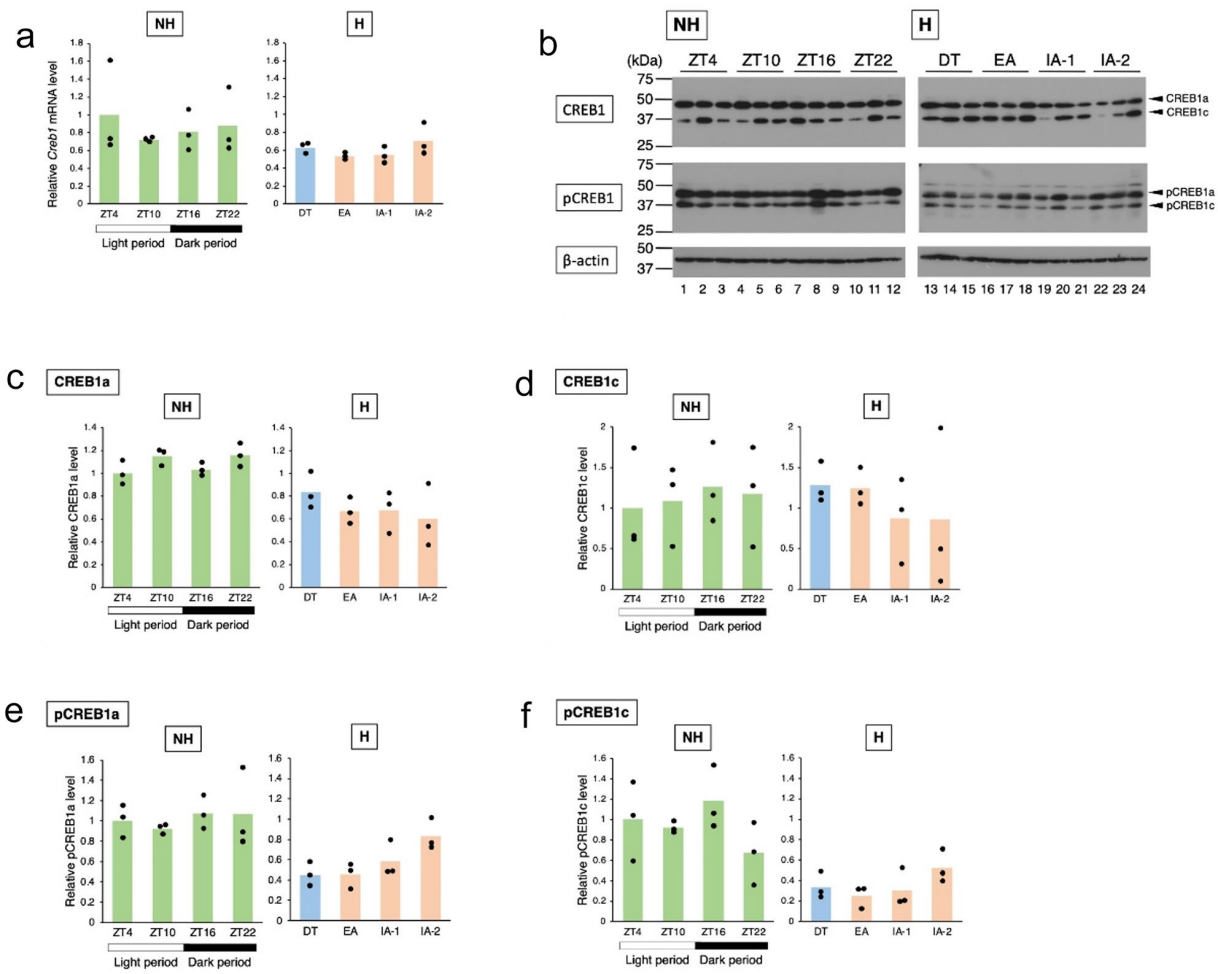
Analysis of *Creb1* expression in chipmunk liver. (**a**) The *Creb1* and *Gapdh* mRNA levels in the non-hibernation (NH) and hibernation (H) seasons were measured by RT-qPCR using chipmunk liver total RNA. qPCR was performed in triplicate. For each time point, three chipmunks were used for quantification. The *Creb1* mRNA levels were normalized to the *Gapdh* mRNA levels and shown relative to the value obtained for ZT4. The data represent the mean of three independent experiments using the liver total RNA from different animals and the scatter plots correspond to the observed values (*n* = 3/time point). There were no statistically significant differences between group means as determined by one-way ANOVA [*p* = 0.785 (NH), *p* = 0.244 (H)]. (**b**) Western blot analysis of CREB1, pCREB1 and β-actin was performed using whole cell extracts prepared from the liver of three chipmunks at each time point. (**c**–**f**) CREB1a, CREB1c, pCREB1a, pCREB1c and β-actin levels were quantified from the western blot analysis images in (**b**) using ImageJ. The CREB1a, CREB1c, pCREB1a and pCREB1c levels were normalized to the corresponding β-actin levels and shown relative to the values obtained for ZT4. The data represent the mean of the results of three samples and the scatter plots correspond to the observed values (*n* = 3/time point). There were no statistically significant differences between group means as determined by one-way ANOVA [(**c**) *p* = 0.111 (CREB1a, NH), *p* = 0.538 (CREB1a, H), (**d**) *p* = 0.945 (CREB1c, NH), *p* = 0.723 (CREB1c, H), (**e**) *p* = 0.831 (pCREB1a, NH), (**f**) *p* = 0.282 (pCREB1c, NH), *p* = 0.213 (pCREB1c, H)]. The one-way ANOVA analysis showed a marginally significant difference [(e) *p* = 0.0413 (pCREB1a, H)], the subsequent Tukey’s HSD test showed no significant difference (Supplementary Table S4). No letters are indicated as no significant differences were observed (one-way ANOVA or Tukey’s HSD test, *p* > 0.05).

The expression of the *Creb1* mRNA in the chipmunk liver was analyzed by RT-qPCR. The qPCR primers were designed on the 5′ side of the insertion sequence in the *Creb1c* mRNA, a sequence common to both the *Creb1a* and *Creb1c* mRNAs, and the total amount of the *Creb1a* + *Creb1c* mRNA was measured (Fig. 4a). The results showed no significant differences by time period for either the non-hibernation or hibernation seasons [one-way ANOVA, *p* = 0.785 (non-hibernation), *p* = 0.244 (hibernation)], but the total *Creb1* mRNA levels were higher in the non-hibernation season than in the hibernation season (Supplementary Fig. S2). Next, to determine whether the ratio of the *Creb1a* mRNA to the *Creb1c* mRNA varied between time periods, the primers were designed on each side of the insertion sequence in the *Creb1c* mRNA (Fig. 3a), and PCR was performed using cDNA synthesized from total RNA prepared from the liver at each time period as a template. The PCR products were subjected to agarose gel electrophoresis, and the respective bands derived from the *Creb1a* and *Creb1c* mRNA were quantified using ImageJ to compare their expression levels (Supplementary Fig. S3). The results showed that the ratio of the *Creb1c* mRNA to the *Creb1a* mRNA increased slightly from ZT4 to ZT22 in the non-hibernation season, but did not change significantly in the hibernation season (one-way ANOVA, *p* = 0.273).

We also examined CREB1 protein levels by Western blot analysis of whole cell extracts prepared from the chipmunk liver using an anti-CREB1 antibody and an anti-phosphorylated CREB1 (S133) antibody, which can detect chipmunk CREB1a and CREB1c phosphorylated at serine 119 (pCREB1a and pCREB1c) (Fig. 4b). The results showed that neither CREB1a nor CREB1c levels varied significantly between time periods in both the non-hibernation and hibernation seasons [one-way ANOVA, *p* = 0.111 (CREB1a, non-hibernation), *p* = 0.538 (CREB1a, hibernation), *p* = 0.945 (CREB1c, non-hibernation), *p* = 0.723 (CREB1c, hibernation)] (Fig. 4c, d). When compared between the non-hibernation and hibernation seasons, there was no significant difference in the CREB1c levels (Student’s *t*-test, *p* = 0.760), but CREB1a levels were reduced in the hibernation season compared to the non-hibernation season (Supplementary Fig. S4). Similarly, neither pCREB1a nor pCREB1c levels varied significantly between time periods in both the non-hibernation and hibernation seasons [one-way ANOVA, *p* = 0.831 (pCREB1a, non-hibernation), *p* = 0.0413 (pCREB1a, hibernation), *p* = 0.282 (pCREB1c, non-hibernation), *p* = 0.213 (pCREB1c, hibernation)], of which, although the one-way ANOVA analysis showed a marginally significant difference for pCREB1a in the hibernation season (*p* = 0.0413), the subsequent Tukey’s test showed no significant difference in pairwise comparisons (Fig. 4e, f). However, both pCREB1a and pCREB1c levels were reduced in the hibernation season compared to the non-hibernation season (Supplementary Fig. S4).

### CREB1a is a candidate activator of *Per1* gene transcription during interbout arousal

**Fig. 5:**
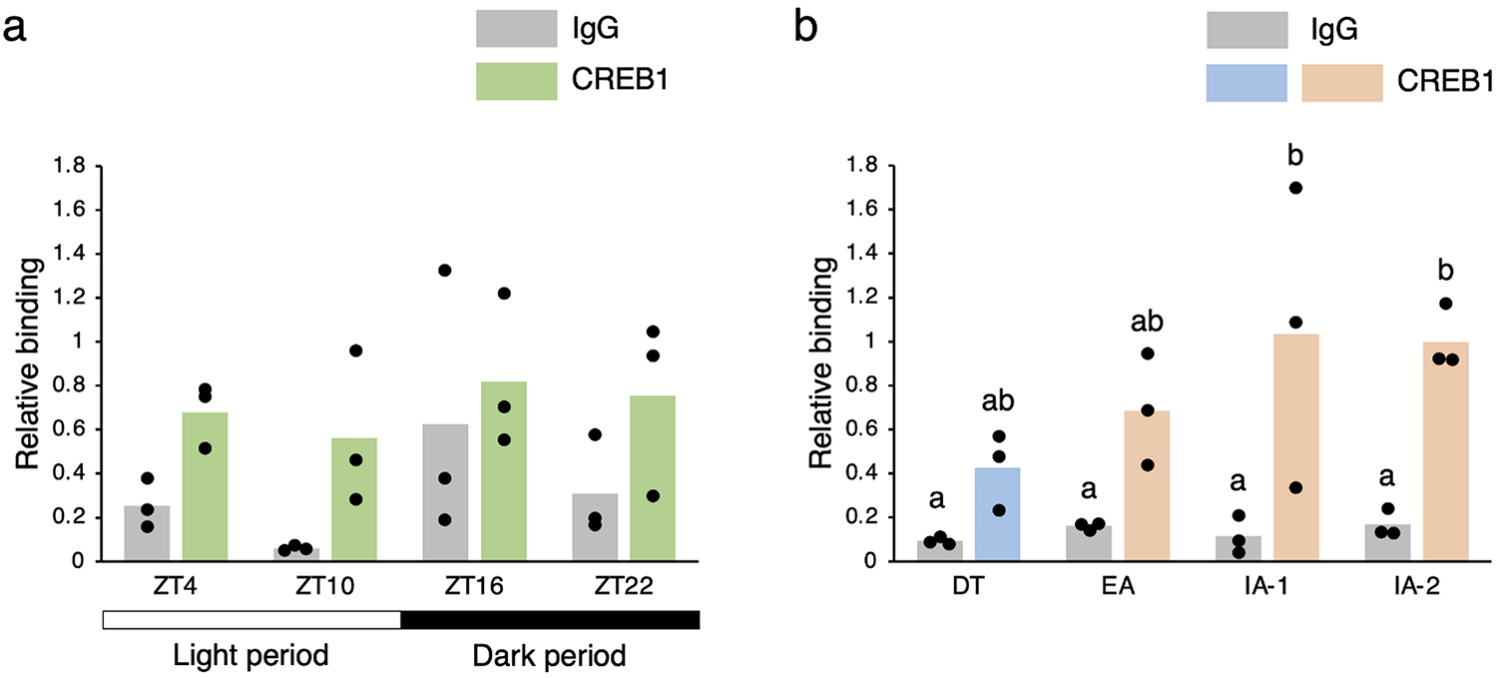
Binding of CREB1a to *Per1* gene promoter during interbout arousal. ChIP analysis was performed using antibodies against CREB1 or histone H3 and normal rabbit IgG with chromatin prepared from the chipmunk liver. qPCR was performed in triplicate using a primer set specific for CRE at – 1698 of the *Per1* gene (Supplementary Fig. S1). Normal IgG served as a negative control. For each time point, three chipmunks were used for quantification. Values were normalized to the corresponding histone H3 values and shown relative to the value obtained for IA-2 with antibodies against CREB1. The data represent the mean and the scatter plots correspond to the observed values (*n* = 3/time point). (**a**) There was no statistically significant difference between group means as determined by one-way ANOVA (*p* = 0.110), and no letters are therefore indicated (one-way ANOVA, *p* > 0.05). (**b**) Different letters indicate significantly different values (Tukey’s HSD test, *p* < 0.05) (Supplementary Table S5).

To determine whether CREB1a is involved in the transcriptional activation of the *Per1* gene in the chipmunk liver, we then analyzed CREB1a binding to the CRE sequence at − 1698 in the promoter region of the *Per1* gene by ChIP using anti-CREB1 antibody (Fig. 5). In the non-hibernation season, the amount of CREB1a bound to the promoter region of the *Per1* gene did not vary significantly (one-way ANOVA, *p* = 0.110). In contrast, in the hibernation season, the amount of CREB1a bound to the promoter region of the *Per1* gene started to increase in EA, although not significantly, and reached a maximum in IA-1. These results, albeit from a small sample size, suggest that CREB1a is likely involved in the transcriptional activation of the *Per1* gene in EA, and that the *Per 1* gene is differentially regulated between the non-hibernation and hibernation seasons.

## Discussion

Circadian rhythms are regulated by TTFL of clock genes. In the chipmunk liver, there is no periodicity in the mRNA expression of the clock gene *Bmal1* in the hibernation season^25^. Therefore, the peripheral circadian clock in the liver is not considered to function in the hibernation season, as TTFL of clock genes is not formed. However, another clock gene *Per2* is activated in the hibernation season, as in the non-hibernation season, by HSF1, which is activated by elevated Tb, and the *Per2* mRNA exhibits periodic fluctuations in the hibernation season^25^. Furthermore, this study showed that in addition to the *Rev-erba* mRNA^25^, the *Per1* mRNA also fluctuates periodically in the hibernation season (Fig. 1). It is noteworthy that both the *Per1* and *Rev-erba* mRNAs fluctuate in a similar phase relationship with the *Per2* mRNA in the hibernation season as in the non-hibernation season. In the non-hibernation season, the *Per1* and *Rev-erba* mRNA have a peak at ZT22 and a trough at ZT10 (Fig. 1), while the *Per2* mRNA levels have a peak at ZT4 and a trough at ZT16^25^. In the hibernation season, the *Per1* mRNA levels increase transiently in EA (Fig. 1), while the *Per2* mRNA levels show a transient increase in IA-1^25^. The *Rev-erba* mRNA shows periodic fluctuations that start to increase in EA and peak in IA-1^25^. Thus, both the *Per1* and *Rev-erba* gene expression is phase-advanced relative to that of the *Per2* gene in both the non-hibernation and hibernation seasons. These results suggest that for a subset of genes, including *Per1*, *Per2* and *Rev-erba*, periodic expression, similar to circadian expression in the non-hibernation season, may be restored during interbout arousal.

HSF1 is required for efficient temperature resetting of circadian rhythms^28–30,38^. However, the observation that in the hibernation season, the *Per1* mRNA increased in EA when the nuclear HSF1 was still low^25^ suggests that factors other than HSF1 are involved in restoring periodic gene expression of the *Per1* mRNA. Notably, BMAL1 was involved in the transcriptional activation of the *Per1* gene at ZT22 in the non-hibernation season, whereas CREB1 was likely involved in the transcriptional activation in EA in the hibernation season (Figs. 2 and 5). In the light-induced resetting of circadian rhythms in SCN, phosphorylated CREB1 is involved in the transcriptional activation of the *Per1* gene^10,35,39–41^. Since transcriptional activation of the *Per1* gene by CREB1a was enhanced by CREB1a phosphorylation (Fig. 3), we performed western blot analysis of CREB1a and pCREB1a using chipmunk nuclear extracts (Supplementary Fig. S5). Neither the amount of CREB1a nor the amount of pCREB1a varied much in the non-hibernation or hibernation season (Supplementary Fig. S5b, c), but the ratio of pCREB1a to CREB1a showed a tendency to increase in EA in the hibernation season (Supplementary Fig. S5d). However, in EA in the hibernation season, pCREB1a did not show a significant increase in binding to the CRE of the *Per1* gene promoter in the ChIP analysis, with large individual differences (Supplementary Fig. S6). In the mouse SCN, the level of CREB binding to the CRE of the *Per1* gene promoter peaked at 60 min after the light stimulation, whereas the level of pCREB binding peaked at 5 min after the light stimulation and rapidly returned to the basal level by 30 min^41^. In view of these results, it is possible that we were not able to detect the binding of pCREB1a to the CRE due to the short binding time of pCREB1a. In cultured NIH3T3 cells and mouse SCN slices, intracellular Ca^2+^ levels increase when culture temperature is reduced^22,42^. Increased intracellular Ca^2+^ levels activate CaMKII, which promotes CLOCK and BMAL1 heterodimer formation via phosphorylation of CLOCK, resulting in transcriptional activation of the *Per1* and *Per2* genes via the E-boxes^43^. Similarly, intracellular Ca^2+^ levels in the chipmunk liver may be increased by hypothermia during deep torpor. Therefore, although BMAL1 binding to the E-box of the *Per1* gene promoter did not increase in EA in the hibernation season (Fig. 2b), CaMK may be activated to phosphorylate CREB1a, and pCREB1a may be involved in the transcriptional activation of the *Per1* gene. Although interpretation should be cautions due to multiple comparisons and small sample size, the results suggest that CREB1a is likely involved in the transcriptional activation of the *Per1* gene in EA, and further studies with larger sample sizes would consolidate the results and help to further elucidate the underlying mechanisms. Moreover, identification of the kinase that regulates CREB1a phosphorylation in chipmunks during hibernation would also be an important avenue for future research.

In the hibernation season, the *Per1* and *Per2* mRNAs levels remained low with little individual variation in torpid animals (DT), and the mRNA rhythms restarted from these aligned levels during interbout arousal, resulting in rhythmic fluctuations in mRNA (Fig. 1)^25^. Using an in vitro culture system of mouse SCN slices, Enoki et al. found that (1) when the incubation temperature was lowered from 35 °C to 22 °C, both the *Bmal1* and *Per2* mRNA rhythms in the mouse SCN neurons persisted, albeit with attenuated amplitudes, and when the temperature was further lowered to 15 °C, no rhythms were detected; (2) when SCN slices incubated at 35 °C were incubated at 15 °C for 4 d and then the culture temperature was returned to 35 °C, the *Per2* mRNA rhythm restarted quickly on day 1 after rewarming at 35 °C, whereas the *Bmal1* mRNA took several days to recover its rhythm^22^. Based on these results, Enoki et al. concluded that when the incubation temperature of SCN slices was lowered from 35 °C to 15 °C, both the *Bmal1* and *Per2* mRNA rhythms were transformed into damped oscillators, with the former eventually suspending at various phases and the latter eventually suspending at a certain phase^22^. In addition, electroencephalographic studies of ground squirrels indicate that deep torpor is entered through sleep^44^. Comparison of liver gene expression profiles between mice and Arctic ground squirrels (AGS) also suggests that the daily sleep-wake cycle in mice shares significant molecular signatures with the deep torpor-interbout arousal cycle in AGS, with deep torpor being similar to sleep^45^. Therefore, given that the *Per1* and *Per2* mRNA levels are already low at ZT16 during the sleep period in the non-hibernation season (Fig. 1)^25^, the restoration of the periodic expression of the *Per1* and *Per2* genes during interbout arousal in the hibernation season may allow their mRNA levels to decrease during the entry into deep torpor, as they do during the entry into sleep in the non-hibernation season, and converge to their respective low levels during deep torpor. It would be intriguing to investigate further whether the partial restoration of periodic expression during interbout arousal is necessary to enter deep torpor, or whether it plays a role in peripheral tissue homeostasis. In future studies, not only a comprehensive comparison of clock gene and clock-controlled gene expression between the non-hibernation and hibernation seasons, but also an analysis of how calcium signaling is regulated and involved in gene expression in the liver in the hibernation season would be valuable for a better understanding of hibernation in mammals.

## Materials and methods

### Animals

Male chipmunks (*Tamias asiaticus*) (age, 2–4 months) purchased from Pet Easy Space (Osaka, Japan) were individually housed and provided with standard rodent chow and water *ad libitum*. They were kept at 23 °C with a 12 h–12 h light-dark photoperiod (light on at 6 AM) during the non-hibernation season (April–September). Zeitgeber time (ZT) 0 (ZT0) corresponds to the time at which the light is on. During the hibernation season (October–March), they were kept under a constant condition of 5 °C in darkness. During the hibernation season, the conditions of the chipmunks were monitored using an infrared activity sensor (O’Hara & Co., Ltd., Tokyo, Japan), and their Tb was recorded by measuring the rectal temperature using a thermistor probe (Thermistor thermometer model KN-91-AD1687-R; Natsume Seisakusho, Tokyo, Japan). Tissue samples from non-hibernating chipmunks (weight, 90–110 g; age, 1–3 years) that were summer-active were obtained between June and August. During the hibernation season, tissue samples were obtained approximately two–four months after the first entry into torpor. Thus, all the chipmunks were acclimated to the photoperiod and temperature conditions of the non-hibernation or hibernation season for a minimum of two months before the experimental samples were collected. Chipmunks in the hibernation season were classified into four categories: hibernating, deeply torpid animals with Tb of 5–7 °C for 2–4 days after entry into deep torpor (DT), early arousal animals whose Tb had not reached 30 °C during re-warming after spontaneous arousal from torpor (EA), interbout awake animals with Tb of 30–35 °C (IA-1) and 35–37 °C (IA-2)^25^. Animals were sacrificed after being deeply anesthetized with isoflurane. Tissues were immediately excised, frozen in liquid nitrogen, and stored at − 80 °C until further use. Hibernating, deeply torpid animals were usually sampled between 12 PM and 4 PM. All protocols were conducted in accordance with the guidelines of the Institutional Animal Care and Use Committee of Kitasato University. All experimental procedures were approved by the same committee. The study was performed in accordance with ARRIVE guidelines.

### Cloning procedures

Total RNA was prepared from chipmunk liver using Isogen (Nippon Gene, catalog no. 311–02501), treated with RNase-free recombinant DNase I (Takara Bio, catalog no. 2270 A), and purified using RNeasy Mini Kit (Qiagen, catalog no. 74104), as per the manufacturer’s instructions. First-strand cDNA was synthesized using PrimeScript 1st strand cDNA Synthesis Kit (Takara Bio, catalog no. 6110 A). Genomic DNA was prepared from the chipmunk liver using Wizard Genomic DNA Purification Kit (Promega, catalog no. A1120).

Chipmunk *Creb1* and *Per1* cDNAs were amplified from chipmunk liver 1st strand cDNA using primers designed based on the corresponding ground squirrel sequences. Amplified fragments were cloned into pBluescript-SK and the sequences were determined. Expression vectors for chipmunk CREB1a, pcDNA3/cmCREB1a, and CREB1c, pcDNA3/cmCREB1c, were constructed by cloning the corresponding cDNA fragment containing the entire coding sequence into pcDNA3.

The genomic DNA fragment containing the promoter region of the chipmunk *Per1* gene was isolated based on the structures of the human and mouse *Per1* genes reported by Hida et al.^33^. PCR was performed using the forward primer designed from the corresponding ground squirrel genomic sequence and reverse primer designed from the corresponding chipmunk cDNA sequence. Amplified fragment was cloned into pBluescript-SK and the sequence was determined. The chipmunk *Per1* gene luciferase reporter plasmid, pCMPER1G/luc, was constructed by cloning the *Per1* gene fragment from − 1885 in the 5ʹ flanking region of exon 1 to + 1664 in the upstream of the translation initiation codon of exon 2 into a promoter-less firefly luciferase expression vector, pGV-B (FUJIFILM, catalog no. 302–02851) (Supplementary Fig. S1).

### Reverse transcription-quantitative polymerase chain reaction (RT-qPCR)

First-strand cDNA was synthesized using DNase I-treated chipmunk liver total RNA and PrimeScript 1st strand cDNA Synthesis Kit (Takara Bio, catalog no. 6110 A). qPCR was carried out using SYBR Green Realtime PCR Master Mix-Plus (Toyobo, catalog no. QPK-211) with the following sets of primers: CREB1-585 F, 5′-AGCAGTGGACAGTATATTGCCA-3′, and CREB1-760R, 5′-TACCTGTCGTCTAGAATCACGG-3′; GAPDH-F1, 5′-GGGTGTGAACCATGAGAAGTATG-3′, and GAPDH-R1, 5′-ACAGTCTTCTGAGTGGCAGTGAT-3′; PER1-1014 F, 5′-CGCCTAACTCCATATGTGACCA-3′, and PER1-1231R, 5′-CGATAGATGGGGTCCTAGAGGA-3′. *Gapdh* was used as the reference gene to normalize RNA quantity.

### Reverse transcription-polymerase chain reaction (RT-PCR)

PCR was carried out using first-strand cDNA synthesized from DNase I-treated chipmunk liver total RNA and PrimeSTAR HS DNA polymerase (Takara Bio, catalog no. R010A) with the following set of primers: CREB1-14 F, 5′-GTGGGGGAGAGAATAAAACTCC-3′, and CREB1-1129R, 5′-CAATTCCACCTTTTACCTGACC-3′. PCR products were subjected to agarose gel electrophoresis, and the bands derived from the *Creb1a* and *Creb1c* mRNAs were quantified using ImageJ.

### Transfection and luciferase assays

HepG2 cells were cultured as previously described^46^. The cells were plated at 5 × 10^4^ cells per 15-mm dish, and after 24 h, were transfected with 100 ng of the chipmunk *Per1* gene luciferase reporter plasmid, pCMPER1G/luc, and 2.5 ng of a *Renilla* luciferase internal control plasmid, pRL-SV40 (Promega, catalog no. E2231), using TransIT-LT1 reagent (Mirus, catalog no. MIR2300). Where indicated, the cells were co-transfected with the indicated amounts of an empty vector, pcDNA3, or an expression vector for the chipmunk CREB1a or CREB1c, pcDNA3/cmCREB1a or pcDNA3/cmCREB1c, or an expression vector for the catalytic subunit of PKA, pFC-PKA (Agilent, catalog no. 219071). After 24 h, luciferase activity was measured using the dual-luciferase reporter assay system (Promega, catalog no. E1910).

### Chromatin immunoprecipitation (ChIP) analysis

ChIP analysis was performed as previously described^46^. All ChIP experiments were performed in triplicate on independent chromatin preparations. The antibodies used were anti-CREB (Cell Signaling Technology: #4820), anti-phospho-CREB (Ser133) (Cell Signaling Technology: #4276), anti-BMAL1 (Santa Cruz Biotechnology: sc-48790), and anti-histone H3 antibody (Abcam: ab1791). Normal rabbit IgG (#2729) was purchased from Cell Signaling Technology. qPCR was carried out using SYBR Green Realtime PCR Master Mix-Plus (Toyobo, catalog no. QPK-211) with the following set of primers: Per1-232 F, 5′-CTTCCTCCGCTTTGACGTCA-3′, and Per1-374R, 5′-CGAAAGGATTCAGCGCTCTAGTA-3′; Per1-1710 F, 5′-TTATTCACTGTGCAAAGCGTCC-3′, and Per1-1875R, 5′-GATTGGCTGGGGATCTCTTCC-3′.

### Western blot analysis

293T cells were grown in Dulbecco modified Eagle medium (DMEM) with 10% fetal bovine serum. The cells were plated at 3 × 10^5^ cells per 35-mm dish, and after 24 h, were transfected with pcDNA3, pcDNA3/cmCREB1a or pcDNA3/cmCREB1c using TransIT-LT1 (Mirus, catalog no. MIR2300)^47^. After 48 h, whole cell extracts, cytoplasmic extracts and nuclear extracts were prepared using Nuclear/Cytosol Fractionation Kit (BioVision, catalog no. 10186-586). Whole cell extracts, cytoplasmic extracts and nuclear extracts of the chipmunk liver were prepared using Nuclear/Cytosol Fractionation Kit (BioVision) (Fig. 3b) or NUN buffer as described by Reinke et al.^47^ (Fig. 4b and Supplementary Fig. S5a). Western blot analysis was carried out as described previously by Fujii et al.^46^. The antibodies used were anti-CREB (Cell Signaling Technology: #4820), anti-phospho-CREB (Ser133) (Cell Signaling Technology: #4276), anti-USF1 (Santa Cruz Biotechnology: sc-390033) and anti-β-actin antibody (Cell Signaling Technology: #4970). After chemiluminescence detection using ImmunoStar LD (FUJIFILM, catalog no. 292-69903) with exposure to X-ray film, quantification of protein bands was carried out using ImageJ.

### Statistical analysis

All experiments were repeated in triplicate. For comparisons between two groups, differences were analyzed using unpaired two-tailed Student’s *t*-test and considered statistically significant at *p* < 0.05. For more than two groups, differences between group means were analyzed using one-way ANOVA and considered significant at *p* < 0.05. Significant results from ANOVA tests were further analyzed using a Tukey’s honestly significant difference (HSD) test to find means that were significantly different from each other (*p* < 0.05).

## Electronic supplementary material

Below is the link to the electronic supplementary material.

Supplementary Material 1

Supplementary Material 2

## Data Availability

The chipmunk DNA sequence data were submitted to DDBJ. The DDBJ accession numbers are LC806972 (*Creb1a*), LC806973 (*Creb1c*), LC806975 (*Per1*), and LC806976 (*Per1* gene from the 5ʹ flanking region of exon 1 to exon 2).
