# Supplementary material for: Periodic expression of Per1 gene is restored in chipmunk liver during interbout arousal in mammalian hibernation
Source: Sci Rep. 2025 Feb 13;15:4403. doi: 10.1038/s41598-025-87299-8 (PMC11825846; doi:10.1038/s41598-025-87299-8)
Supplement: Supplementary file 1 — Supplementary Material 1 [file 41598_2025_87299_MOESM1_ESM.pdf]

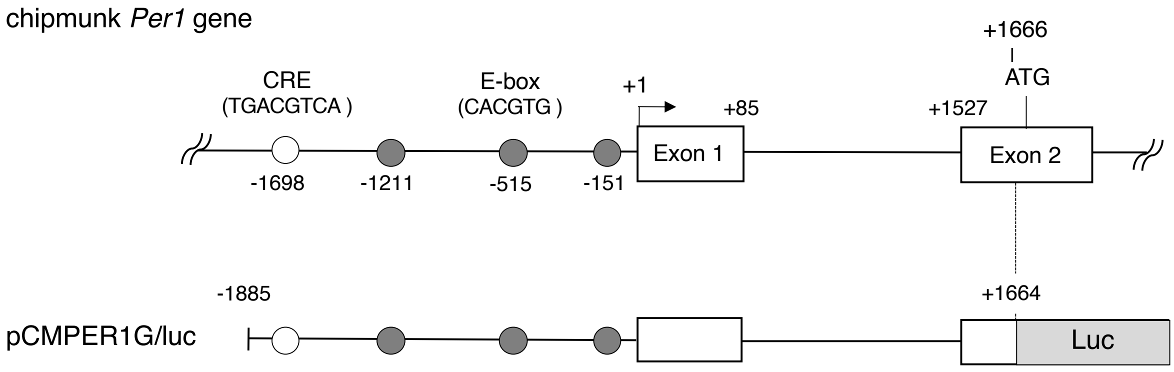


**Supplementary Fig. S1. Structure of chipmunk *Per1* gene.** Schematic diagrams of the chipmunk *Per1* gene from the 5′ flanking region of exon 1 to exon 2 (top) and the *Per1* gene luciferase reporter plasmid pCMPER1G/luc (bottom) are shown. The transcription start site is indicated as +1. The positions of the CRE and E-boxes are indicated by white and black circles, respectively.

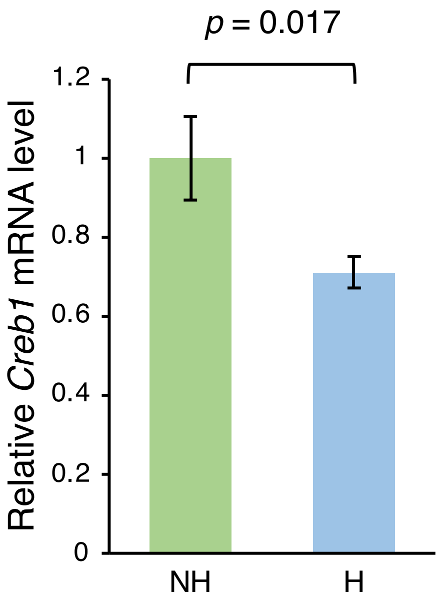


**Supplementary Fig. S2. Comparison of *Creb1* mRNA levels between non-hibernation and hibernation seasons.** The *Creb1* mRNA levels normalized to the *Gapdh* mRNA levels in Fig. 4a were compared between the non-hibernation (NH) and hibernation (H) seasons, and the results are shown as mean ± SEM (n = 12). Statistical differences were examined by unpaired two-tailed Student's *t*-test.

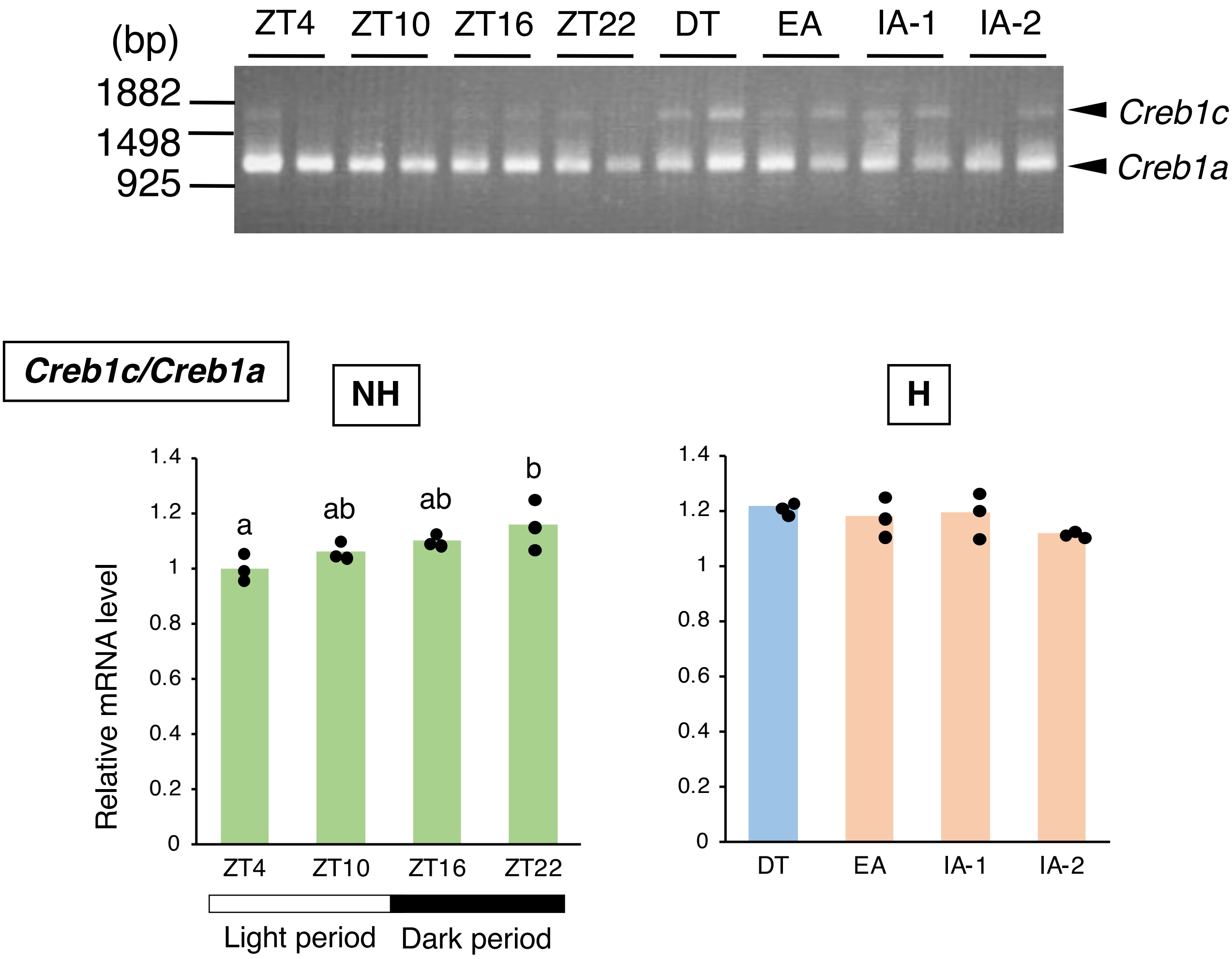


**Supplementary Fig. S3. Analysis of the ratio of *Creb1c* mRNA to *Creb1a* mRNA.** PCR was performed using chipmunk liver cDNA with primers designed on each side of the insertion sequence in the *Creb1c* mRNA, and the PCR products were subjected to agarose gel electrophoresis (upper panel). The respective bands derived from the *Creb1a* and *Creb1c* mRNA were quantified using ImageJ to compare their expression levels, and the results are shown relative to the value obtained for ZT4 (lower panel). The data represent the mean of the results of three samples and the scatter plots correspond to the observed values (n = 3/time point). In the lower left panel (NH), different letters indicate significantly different values [Tukey's HSD test, $p < 0.05$] (Supplementary Table S6). In the lower right panel (H), there was no statistically significant difference between group means as determined by one-way ANOVA [$p = 0.273$], and no letters are therefore indicated (one-way ANOVA, $p > 0.05$).

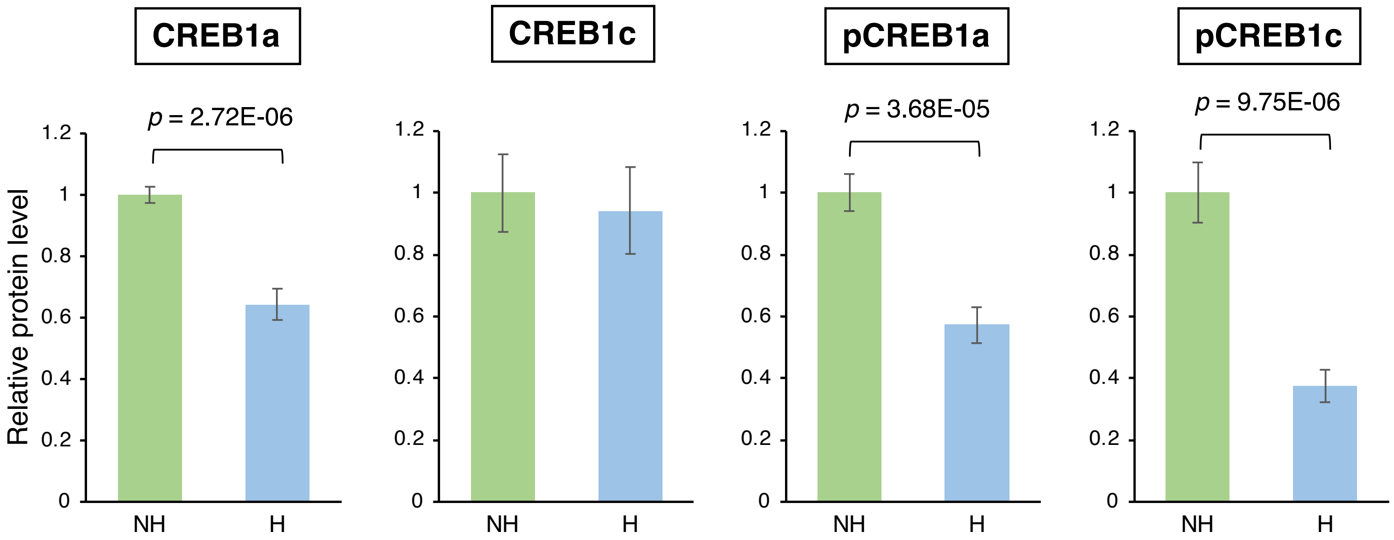


**Supplementary Fig. S4. Comparison of CREB1 protein levels between non-hibernation and hibernation seasons.** CREB1a, CREB1c, pCREB1a and pCREB1c levels normalized to those of β-actin in Figs. 4c-f were compared between the non-hibernation (NH) and hibernation (H) seasons, and the results are shown as mean ± SEM (n = 12). Statistical differences were examined by unpaired two-tailed Student's *t*-test. For CREB1c, there was no statistically significant difference between group means ($p = 0.760$).

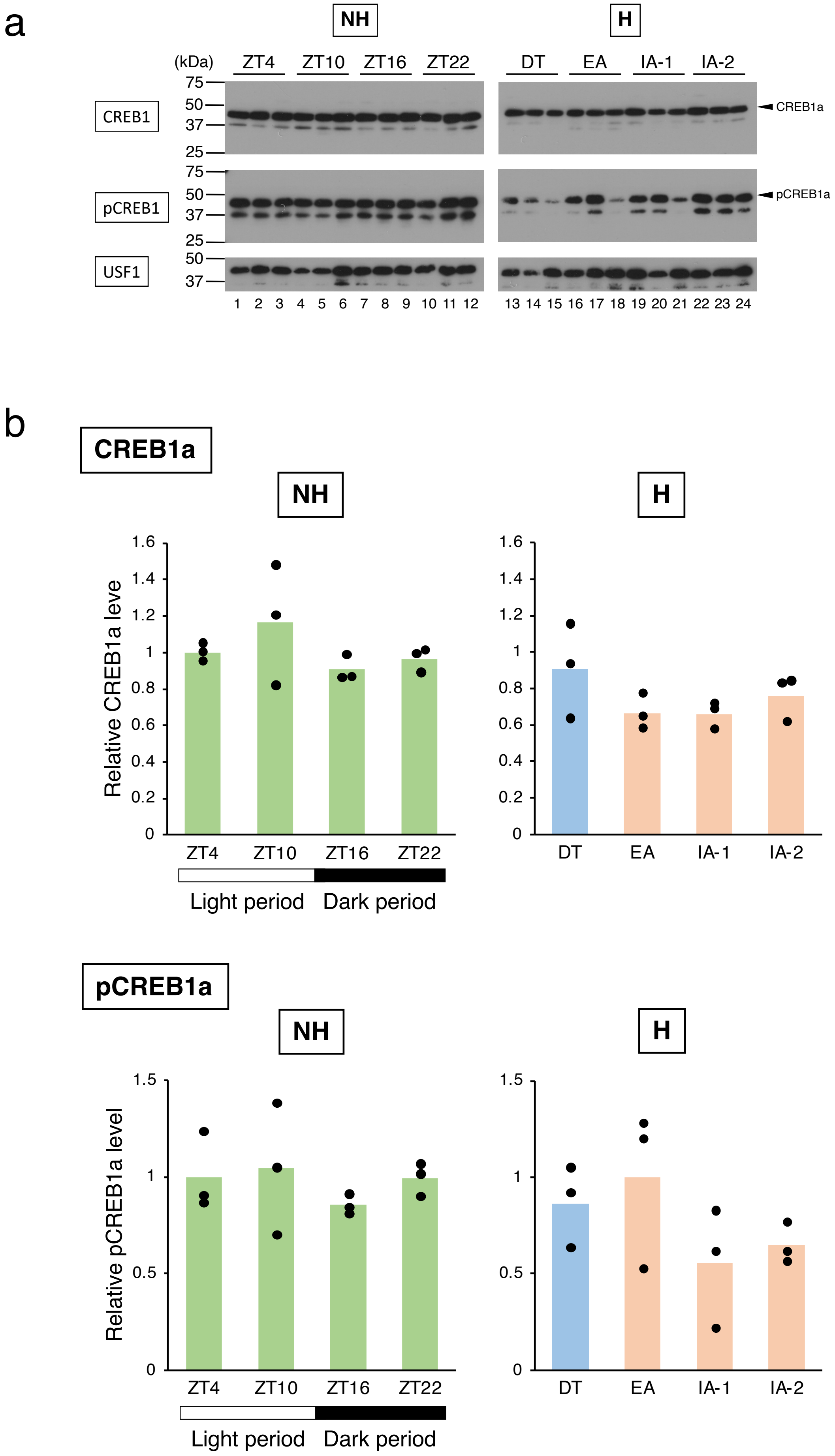

a
NH
H
(kDa)
ZT4
ZT10
ZT16
ZT22
DT
EA
IA-1
IA-2
75
50
37
25
CREB1
pCREB1
USF1
CREB1a
pCREB1a
1 2 3 4 5 6 7 8 9 10 11 12
13 14 15 16 17 18 19 20 21 22 23 24
b
CREB1a
NH
H
Relative CREB1a leve
1.6
1.4
1.2
1
0.8
0.6
0.4
0.2
0
ZT4 ZT10 ZT16 ZT22
DT EA IA-1 IA-2
Light period
Dark period
pCREB1a
NH
H
Relative pCREB1a level
1.5
1
0.5
0
ZT4 ZT10 ZT16 ZT22
DT EA IA-1 IA-2
Light period
Dark period

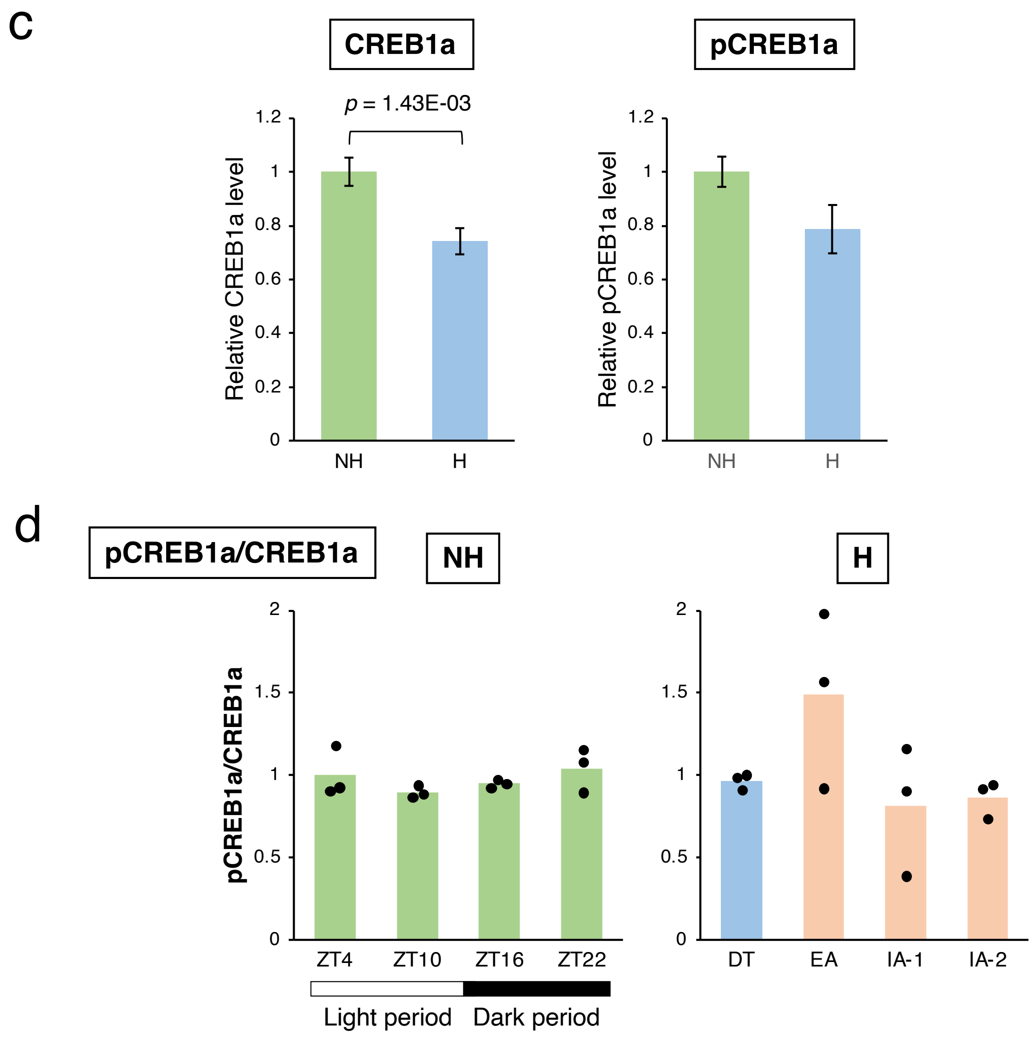


**Supplementary Fig. S5. Western blot analysis of nuclear CREB1a and pCREB1a.** (**a**) Western blot analysis of CREB1 was performed using nuclear extracts prepared from the liver of three chipmunks at each time point. (**b**) CREB1a, pCREB1a and USF1 levels in (**a**) were quantified by ImageJ. The CREB1a and pCREB1a levels were normalized to the corresponding USF1 levels and shown relative to the values obtained for ZT4. The data represent the mean of the results of three samples and the scatter plots correspond to the observed values (n = 3/time point). There were no statistically significant differences between group means as determined by one-way ANOVA [$p$ = 0.379 (CREB1a, non-hibernation), $p$ = 0.257 (CREB1a, hibernation), $p$ = 0.710 (pCREB1a, non-hibernation), $p$ = 0.275 (pCREB1a, hibernation)]. (**c**) Based on the results of (**b**), CREB1a and pCREB1a levels were compared between the non-hibernation (NH) and hibernation (H) seasons, and the results are shown as mean ± SEM (n = 12). Statistical differences were examined by unpaired two-tailed Student's $t$-test [$p$ = 0.0575 (pCREB1a)]. (**d**) Based on the results of (**b**), the ratios of pCREB1a to CREB1a are shown relative to the value obtained for ZT4. The data represent the mean and the scatter plots correspond to the observed values (n = 3). There were no statistically significant differences between group means as determined by one-way ANOVA [$p$ = 0.413 (non-hibernation), $p$ = 0.131 (hibernation)], and no letters are therefore indicated (one-way ANOVA, $p > 0.05$).

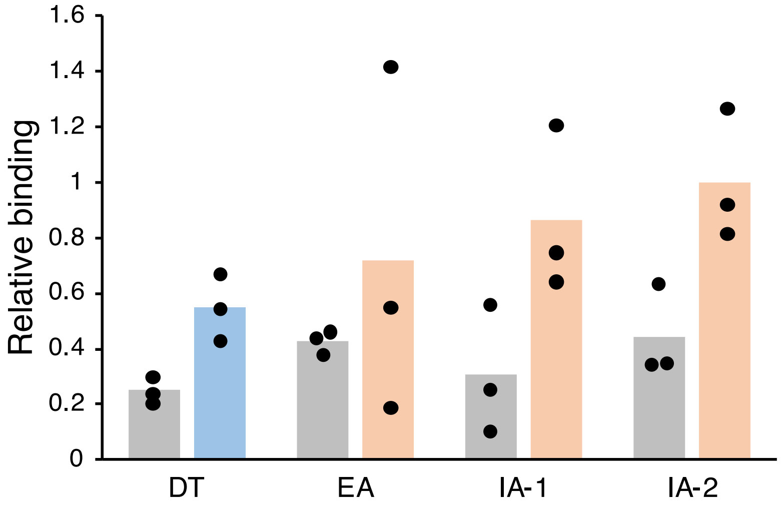


**Supplementary Fig. S6. ChIP analysis of pCREB1a binding to *Per1* gene promoter.** ChIP analysis was performed with chromatin from the liver of three chipmunks at each time point in the hibernation season using antibodies against phospho-CREB (Ser133) or histone H3 and normal rabbit IgG as in Fig. 5. Values were normalized to the corresponding histone H3 values and shown relative to the value obtained for IA-2 with antibodies against phospho-CREB (Ser133). Although the one-way ANOVA analysis showed a marginally significant difference ($p$ = 0.0492), there were no significant differences between any pair of group means as determined by Tukey's honestly significant difference (HSD) test, and no letters are therefore indicated (Tukey's HSD test, $p > 0.05$) (Supplementary Table S7).

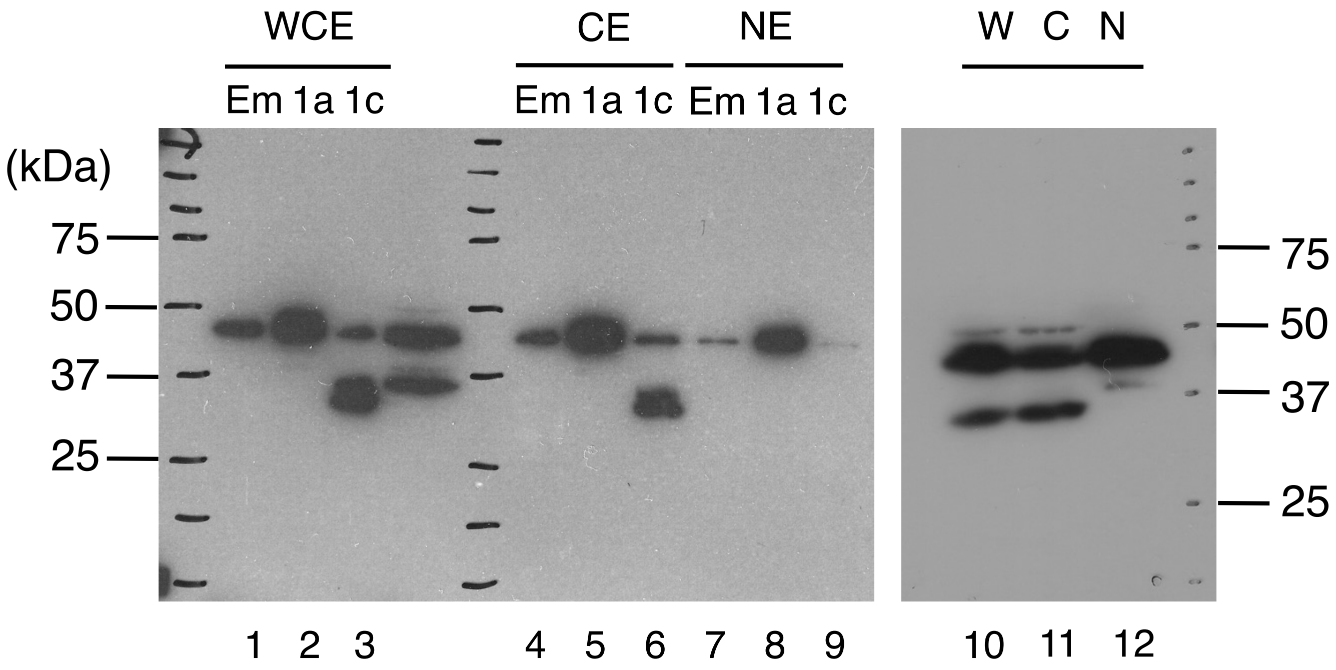


**Supplementary Fig. S7. Full-length blots of Fig. 3b.**

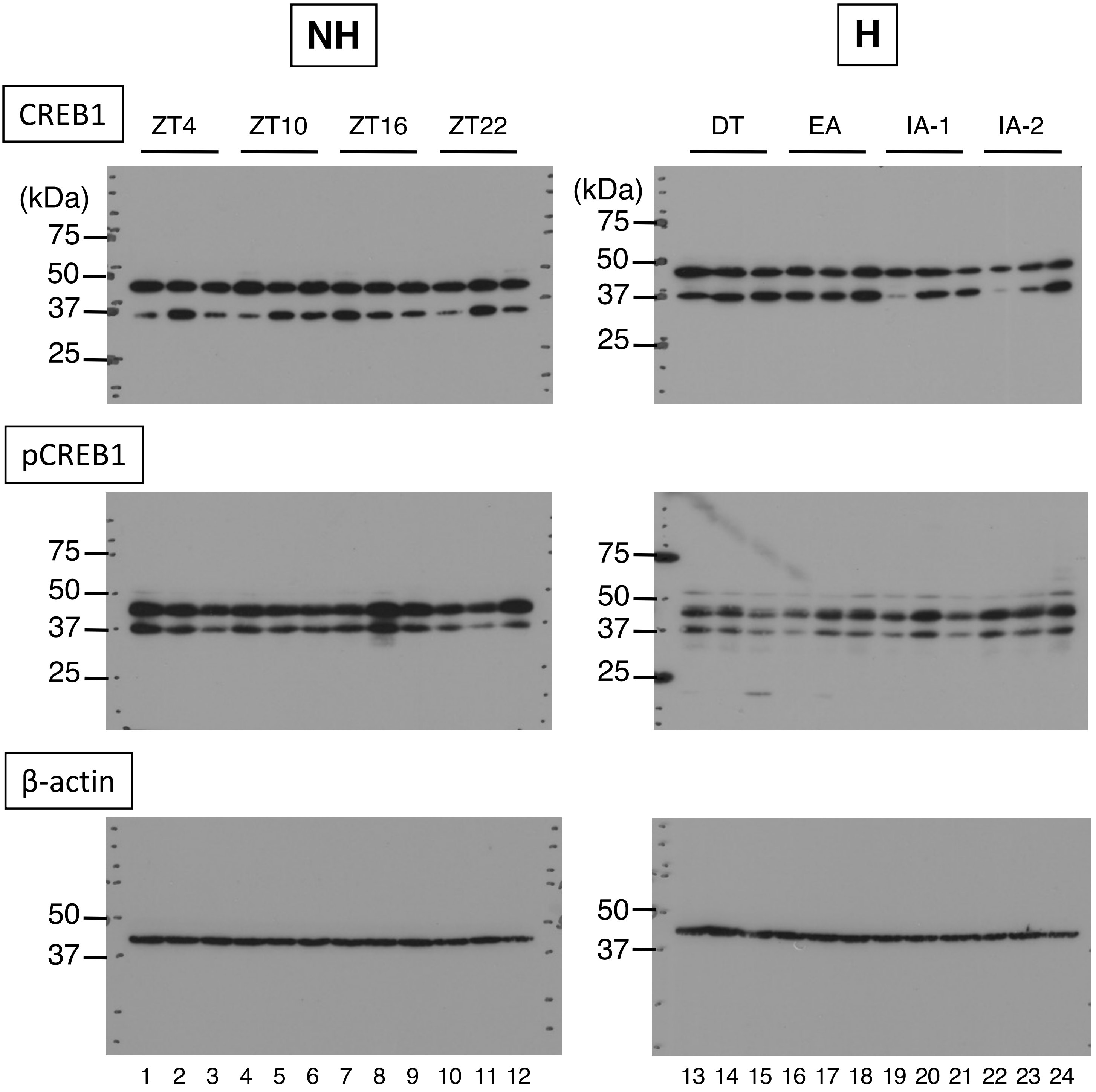


**Supplementary Fig. S8. Full-length blots of Fig. 4b.**

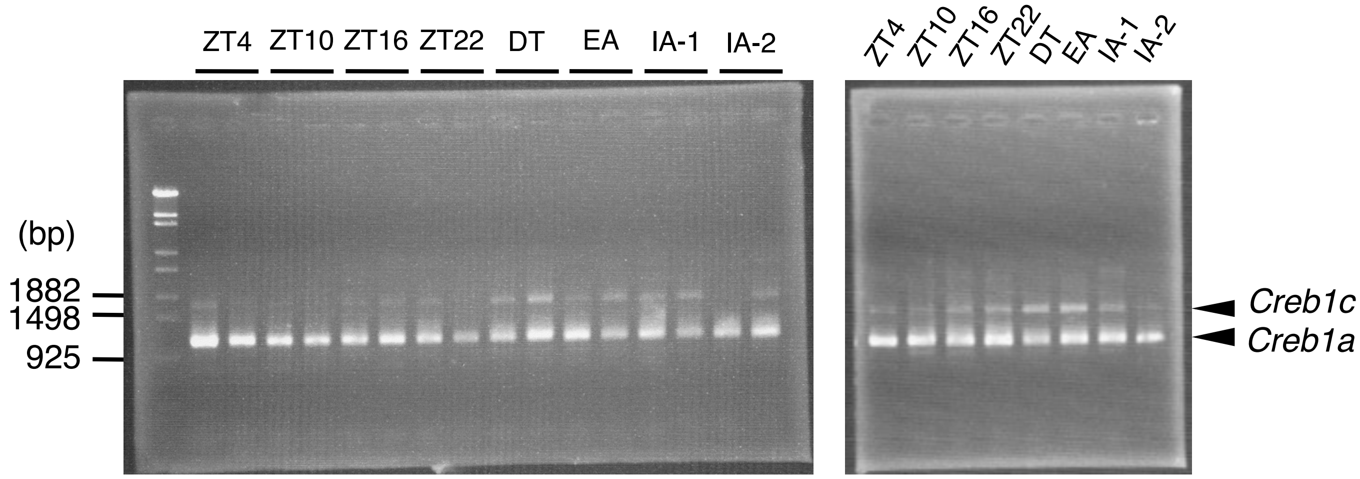


**Supplementary Fig. S9. Full-length gels of Supplementary Fig. S3.**

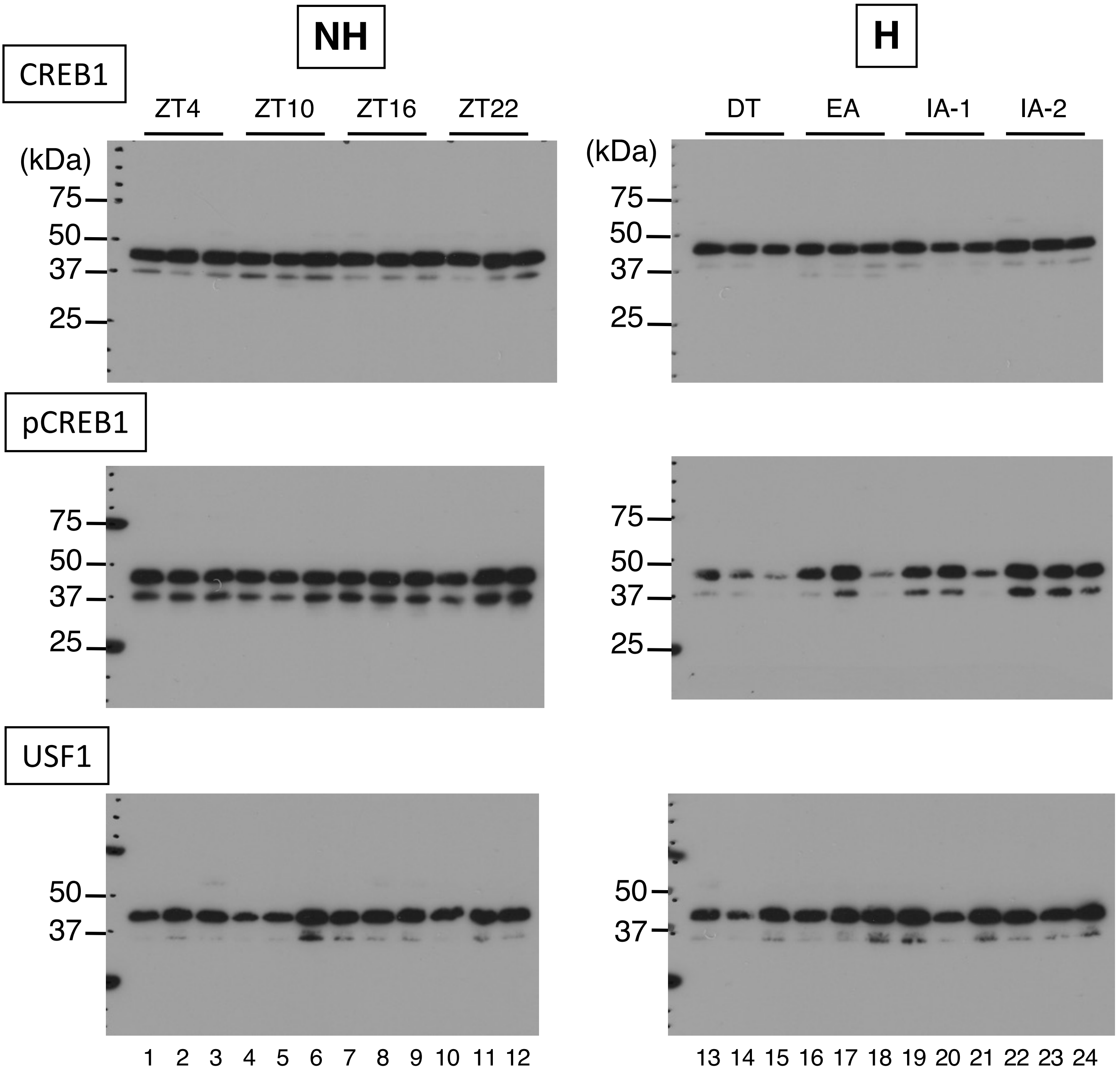


**Supplementary Fig. S10. Full-length blots of Supplementary Fig. S5a.**
