# Supplementary material for: Periodic expression of Per1 gene is restored in chipmunk liver during interbout arousal in mammalian hibernation
Source: Sci Rep. 2025 Feb 13;15:4403. doi: 10.1038/s41598-025-87299-8 (PMC11825846; doi:10.1038/s41598-025-87299-8)
Supplement: Supplementary file 2 — Supplementary Material 2 [file 41598_2025_87299_MOESM2_ESM.pdf]

Supplementary Table S1. Results of Tukey's HSD test for Figures 1a and 1b

Figure 1a

	ZT4	ZT10	ZT16	ZT22
ZT4		0.006	0.008	0.364
ZT10			0.996	< 0.001
ZT16				0.001
ZT22				

Figure 1b

	DT	EA	IA-1	IA-2
DT		< 0.001	0.002	0.015
EA			0.210	< 0.001
IA-1				< 0.001
IA-2				

P values for Tukey's HSD test results are shown. Values below 0.05 are marked in red. The *p* values for one-way ANOVA were 0.000332 for Figure 1a and 8.02E-06 for Figure 1b.

Supplementary Table S2. Results of Tukey's HSD test for Figures 2a and 2b

Figure 2a

		ZT4		ZT10		ZT16		ZT22	
		IgG	BMAL1	IgG	BMAL1	IgG	BMAL1	IgG	BMAL1
ZT4	IgG		0.639	0.997	1.000	1.000	0.923	1.000	0.001
	BMAL1			0.288	0.863	0.817	0.999	0.449	0.036
ZT10	IgG				0.953	0.971	0.601	1.000	< 0.001
	BMAL1					1.000	0.993	0.994	0.003
ZT16	IgG						0.985	0.997	0.002
	BMAL1							0.787	0.012
ZT22	IgG								< 0.001
	BMAL1								

Figure 2b

		DT		EA		IA-1		IA-2	
		IgG	BMAL1	IgG	BMAL1	IgG	BMAL1	IgG	BMAL1
DT	IgG		0.996	1.000	1.000	1.000	0.034	1.000	0.026
	BMAL1			0.966	1.000	0.999	0.122	0.998	0.098
EA	IgG				0.999	1.000	0.019	1.000	0.015
	BMAL1					1.000	0.051	1.000	0.040
IA-1	IgG						0.044	1.000	0.035
	BMAL1							0.038	1.000
IA-2	IgG								0.030
	BMAL1								

P values for Tukey's HSD test results are shown. Values below 0.05 are marked in red. The *p* values for one-way ANOVA were 0.000425 for Figure 2a and 0.00193 for Figure 2b.

Supplementary Table S3. Results of Tukey's HSD test for Figure 3c

Figure 3c

		PKA-			PKA+		
		pcDNA3	CREB1a	CREB1c	pcDNA3	CREB1a	CREB1c
PKA-	pcDNA3		< 0.001	1.000	0.038	< 0.001	0.766
	CREB1a			< 0.001	0.200	0.068	0.005
	CREB1c				0.039	< 0.001	0.780
PKA+	pcDNA3					0.001	0.306
	CREB1a						< 0.001
	CREB1c						

P values for Tukey's HSD test results are shown. Values below 0.05 are marked in red. The *p* value for one-way ANOVA was 3.86E-06.

Supplementary Table S4. Results of Tukey's HSD test for Figure 4e

Figure 4e: pCREB1a (H)

	DT	EA	IA-1	IA-2
DT		1.000	0.703	0.054
EA			0.705	0.054
IA-1				0.244
IA-2				

P values for Tukey's HSD test results are shown. The *p* value for one-way ANOVA was 0.0413.

Supplementary Table S5. Results of Tukey's HSD test for Figure 5b

Figure 5b

		DT		EA		IA-1		IA-2	
		IgG	CREB1	IgG	CREB1	IgG	CREB1	IgG	CREB1
DT	IgG		0.798	1.000	0.202	1.000	0.011	1.000	0.015
	CREB1			0.922	0.929	0.845	0.178	0.930	0.227
EA	IgG				0.319	1.000	0.020	1.000	0.027
	CREB1					0.236	0.757	0.333	0.835
IA-1	IgG						0.014	1.000	0.018
	CREB1							0.021	1.000
IA-2	IgG								0.029
	CREB1								

P values for Tukey's HSD test results are shown. Values below 0.05 are marked in red. The *p* value for one-way ANOVA was 0.00104.

Supplementary Table S6. Results of Tukey's HSD test for Supplementary Figure S3 (NH)

Supplementary Figure S3 (NH)

	ZT4	ZT10	ZT16	ZT22
ZT4		0.551	0.196	0.032
ZT10			0.826	0.215
ZT16				0.590
ZT22				

P values for Tukey's HSD test results are shown. Values below 0.05 are marked in red. The *p* value for one-way ANOVA was 0.0421.

Supplementary Table S7. Results of Tukey's HSD test for Supplementary Figure S6

Supplementary Figure S6

		DT		EA		IA-1		IA-2	
		IgG	pCREB	IgG	pCREB	IgG	pCREB	IgG	pCREB
DT	IgG		0.892	0.993	0.499	1.000	0.207	0.988	0.076
	pCREB			0.993	0.994	0.962	0.859	0.997	0.540
EA	IgG				0.900	0.999	0.574	1.000	0.273
	pCREB					0.646	0.998	0.924	0.916
IA-1	IgG						0.303	0.999	0.119
	pCREB							0.618	0.999
IA-2	IgG								0.304
	pCREB								

P values for Tukey's HSD test results are shown. The *p* value for one-way ANOVA was 0.0492.
